# Expressive amusia and aphasia: the story of Maurice Ravel

**DOI:** 10.1590/1980-5764-DN-2023-0108

**Published:** 2024-06-24

**Authors:** Sultan Darvesh, Meghan Kirsten Cash, Earl Martin, Eliasz Engelhardt

**Affiliations:** 1 Dalhousie University, Department of Medical Neuroscience, Halifax, Nova Scotia, Canada.; 2 Dalhousie University, Department of Medicine (Geriatric Medicine & Neurology), Halifax, Nova Scotia, Canada.; 3 Mount St. Vincent University, Department of Chemistry and Physics, Halifax, Nova Scotia, Canada.; 4 Universidade Federal do Rio de Janeiro, Instituto de Neurologia Deolindo Couto e Instituto de Psiquiatria, Rio de Janeiro RJ, Brazil.

**Keywords:** Maurice Ravel, Aphasia, Apraxia, Auditory Perceptual Disorders, Maurice Ravel, Afasia, Apraxia, Transtornos da Percepção Auditiva

## Abstract

The French composer, Maurice Ravel, at the peak of his career, showed signs of a progressive disorder that affected his ability to function with verbal and musical language, as noted by the neurologist Théophile Alajouanine. The worsening of the disease led to a craniotomy, performed in 1937, which failed to reveal the cause of his illness, and he died shortly thereafter. A lack of post-mortem neuropathological evidence precluded a definitive diagnosis of the illness, which remained enigmatic. Speculations about the precise diagnosis of Ravel's neurological disease have been largely based on Alajouanine's observations, which included aphasia and amusia, mostly expressive, and ideomotor apraxia, while musical judgement, taste, and memory remained relatively intact, implying different neuroanatomical substrates. A possible subform of frontotemporal lobar degeneration complex was the diagnostic suggestion of many authors. His untimely death deprived the world of this remarkable musician, and the music that remained trapped in his mind.

## RESUMO

O compositor francês Maurice Ravel, no ápice de sua carreira, mostrou sinais de uma desordem progressiva que afetou sua capacidade de funcionar com linguagem verbal e musical, como notado pelo neurologista Théophile Alajouanine. O agravamento de sua condição levou a uma craniotomia, efetuada em 1937, que deixou de revelar a causa de sua doença, tendo ele falecido pouco depois. A ausência de evidência neuropatológica pós-morte impediu o diagnóstico definitivo da doença, que permaneceu enigmático. Especulações sobre o diagnóstico preciso da doença neurológica de Ravel foram baseadas sobretudo nas observações de Alajouanine, que compreendiam afasia e amusia, predominantemente expressiva, e também apraxia, enquanto o julgamento, gosto e memória musicais permaneceram relativamente intactos, implicando diferentes substratos neuroanatômicos. A possibilidade de uma subforma do complexo da degenearação lobar frontotemporal foi a sugestão diagnóstica de muitos autores. A sua morte prematura privou o mundo desse notável músico e da música que permaneceu presa em sua mente.

## INTRODUCTION

**Figure 1:**
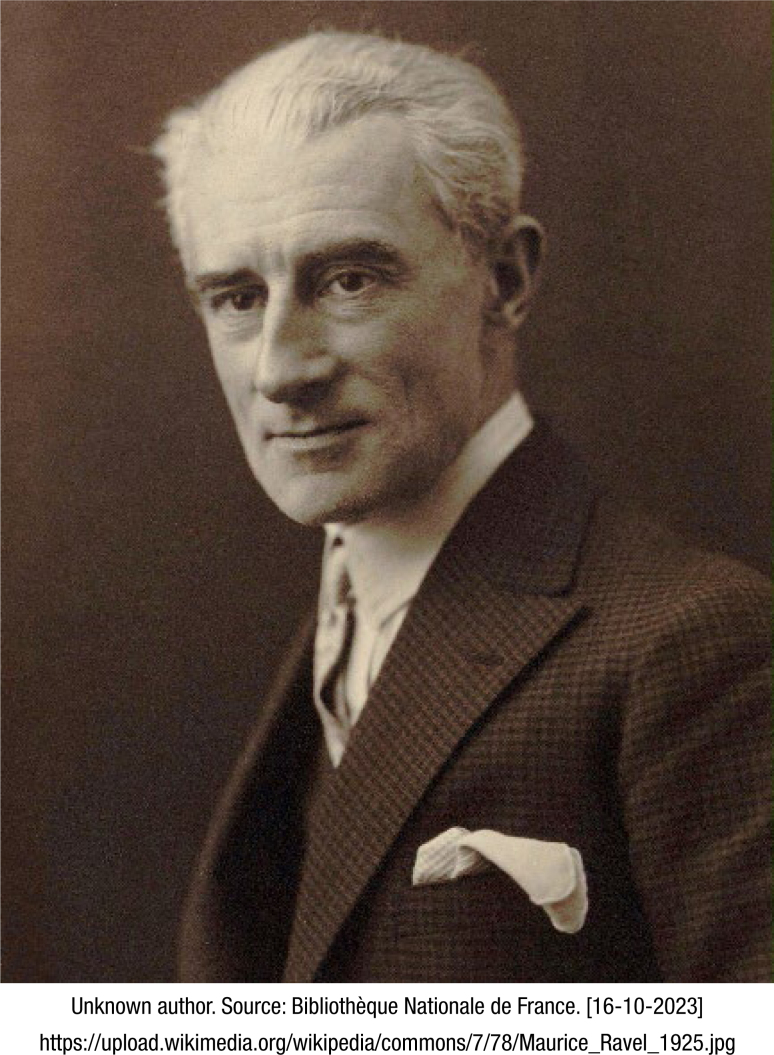
Maurice Ravel (1925).

Maurice Ravel (1875–1937), the celebrated composer, pianist, and conductor of the French Impressionist period, was born on March 7^th^, 1875, in Ciboure, France, to a Basque mother, Marie, and a Swiss father, Joseph, an engineer and accomplished pianist. The family encouraged development in the arts^1^. Ravel began his musical education at the age of seven with Henry Ghys as his piano teacher, and subsequently attended the Paris Conservatory of Music^2^ (Figure 1).

Unknown author. Source: Bibliothèque Nationale de France. [16-10-2023] https://upload.wikimedia.org/wikipedia/commons/7/78/Maurice_Ravel_1925.jpg

For years Ravel was subject to psychiatric symptoms^3^, among them insomnia, fatigue, and depression, and in 1912 was diagnosed with "incipient neurasthenia"^1^. He served in the French Army during WWI but was discharged in 1917 because of poor health. His problems with anxiety and depression were undoubtedly exacerbated by the death of his mother in that year^4^.

Ravel was of slight stature and had a disproportionately large head. He took long walks and swam for exercise. He was a chain smoker who enjoyed strong coffee, large wines, and hot spices^3,5^.

Despite health issues, Ravel's middle years were productive. He developed numerous compositions, enjoying great success as a composer and conductor in tours of Europe and North America^6,7^. However, by 1931 he developed major depression and was admitted to a Swiss clinic^8^. In retrospect, it was suggested that signs of Ravel's developing symptoms had emerged as early as 1927^3,9,10^. Around this time, Ravel was having difficulty finding and writing words as well as writing music, such that by 1931 he described himself as being "nearly finished"^3^.

Ravel, a swimmer, also reported early in 1932 that he had lost the capacity for this favorite exercise, due to an inability to co-ordinate limb movements^3,8^. Later that year, he was in a taxi accident in Paris and suffered facial injuries (right superciliary arch and right jaw region, left jaw region, and luxation of the nasal cartilage), but there was no report of loss of consciousness. Additionally, there were severe thoracic injuries, with right-sided pleural effusion (diagnosed as hemothorax by the attending physician), and fever in the following days^3,7^. Treatment following the accident involved acupuncture and hypnosis^2,3^. No new musical compositions were produced after the 1932 accident, and Ravel was seldom seen in public again^3,10^. By 1933, Ravel was examined by a notable neurologist in Paris, Théophile Alajouanine, who followed up on him until 1936^3,11,12^.

### Alajouanine's examination

Alajouanine performed the initial neurological examination with neuropsychological items in 1933. The neurologist, having musical abilities himself, added the piano to the collection of assessment tools to help evaluate the various components of Ravel's impairments. He also engaged the services of a favorite pupil of Ravel's, Manuel Rosenthal, to assist in a series of tests involving music^3,11–13^.

#### The neurological and neuropsychological examination

Alajouanine's synthetic description begins with the statement: "Maurice Ravel was struck down by an aphasia." And follows: "…it is a Wernicke aphasia of moderate intensity, oral and written [verbal] language are diffusely impaired, but moderately so, without any noticeable intellectual weakening… with an ideomotor apractic component…"^11^. He also affirmed that there was no paralysis nor hemianopia. He then stated that Ravel's writing was very faulty, mainly due to apraxia, and that comprehension was much better than oral or written abilities, while memory, judgment, affectivity, and aesthetic taste did not show impairment^11^. Regarding musical language, he described: "…musical language is still more impaired [relative to verbal]…[with a] remarkable discrepancy between a loss of musical expression (written or instrumental), and musical thinking, which is comparatively well preserved…"^11^.

A special assessment of musical function followed.

#### Musical assessment

The detailed musical assessment was comprised of tests to examine Ravel's performance in diverse musical components (Box 1)^11^.

Box 1 **Musical assessment of Ravel performed by Alajouanine, assisted by Rosenthal (1933)^11^.**

The tested musical components comprised ‘musical thinking’, ‘tune recognition and note reading’, ‘piano playing’, ‘musical writing’, ‘singing’, and ‘musical listening’.

**Musical thinking.** Ravel could recognize pieces of music appropriately, and was able to appreciate rhythm and style, as he recognized errors and wanted the piece to be played properly when several parts of *Tombeau de Couperin* were played with minor errors. He identified the error, when one of two exactly similar bars was omitted from the beginning of *La Pavane de Ma Mere-L'Oye*, and was able to explain the link between the two bars. Ravel recognized that Alajouanine's piano was out of tune by playing two separate notes, demonstrating a lack of harmony between the notes.

**Tune recognition and note reading.** His recognition of tunes was generally good and prompt. Ravel recognized immediately most of the works he knew, and perfectly his own works. However, there was great difficulty reading musical notes, including solfeggio [ability to read and sing music at sight].

**Piano playing.** It was almost impossible after reading, and beside the difficulty in reading, he had to search for the location of notes on the keyboard and often misplaced notes. Ravel could play by heart pieces of his own composition. He could play the first seven or eight bars of *Le Tombeau de Couperin* almost perfectly and transpose to the lower tierce without error but was unable to finish. There was much greater difficulty with unknown pieces, as he could not play more than two or three notes of a piece by Domenico Scarlatti.

**Musical writing.** There was difficulty in writing music, although this ability was better preserved than verbal writing. He wrote dictated notes slowly and with numerous errors, and copying was almost impossible and required enormous effort. Writing a portion of one of his compositions, by heart, though difficult and slow, was better performed than other tests.

**Singing.** He was able to sing, by heart, some of his own compositions, but only if the first note(s) were given.

**Music listening.** Ravel attended concerts, and expressed criticism or described the musical pleasure he felt. The artistic sensibility and judgment did not seem to be altered, as his repeated admiration for the romantic composer Weber showed.

Alajouanine followed the musician over a two-year period, with the tests indicating progressive deficits of verbal and musical languages, mostly expressive, such that it became impossible for Ravel to write, compose, and play music. Contrastingly, appreciation, judgement, and memory were relatively unimpaired. Ravel's symptoms progressively worsened from 1933 to 1937, with increasing loss of the ability to read and write music, as well as to conduct an orchestra. In 1935 he was said to be pale faced, without energy, and emaciated. By Autumn 1937 and unable to express music, Ravel lamented, "*Et puis, j'avais encore tant de musique dans la tête"* ^10,12^.

### Alajouanine's summary and diagnosis

Alajouanine summarized: "…because of aphasia, and…of a simultaneous apraxia, musical reading, piano playing, use of musical signs is much more impaired than expression and recognition of musical themes. Severe disturbance of realization, and difficulty of expressing a relatively preserved musical thinking…"^11^.

Regarding the diagnosis, even ten years after Ravel's death, Alajouanine was unable to conclude otherwise: "The cause remains imprecise, it is however located, considering the bilateral ventricular dilatation, among the pictures of the cerebral atrophies, although different from a true Pick's disease"^10,11^.

### The surgery

Several diagnostic possibilities were considered, such as hydrocephalus, brain tumor, subdural hematoma, and unknown neurodegenerative condition^3,9,10^. A pneumoencephalography (PEG) examination was performed by Thierry de Martel that revealed hydrocephalus^2,3,10^. Intervention was recommended by some, but the neurosurgeon de Martel refused to perform and advised against the surgery^2,10,12^. The neurosurgeon Professor Clovis Vincent repeated the PEG, and recommended the intervention, authorized by the composer's brother. The operation, with a preoperatory diagnosis of "ventricular dilatation", was performed by Vincent, without anesthesia, "as customary for these kinds of interventions in the brain", on December 17^th^, 1937 (Protocol — Box 2)^3,4,9,14^. Ravel endured it calmly and lapsed into sleepiness during the intervention. He awoke the next day and pronounced some incoherent words. A day later he recovered consciousness for a short time, lapsed again into a sleeping state, and became comatose some hours later. He perished on December 28^th^, 1937, at age 62 years^2,4,9,14,15^. Autopsy was not permitted by the family^3,10,14^.

Box 2 **Protocol of Prof. Clovis Vincent's surgical report describing the intervention^3,4,9,14^.**

"Right front flap, with frontotemporal basis. Scalp: Complete separation of the bone; Vertical saw; Dura tacked, but not fastened. Immediate hanging up of the dura, by the vessels. Transversal aperture of the dura. Slack brain, without actual softening in the area displayed. Gyri separated by oedema, but not atrophied. Puncture of the right lateral ventricle: cerebral fluid escapes only on pressure. Injection of 20 cc of water, empties immediately. Multiple attempts. Finally, the injection hole is closed by coagulation; the dura is left open. Reposition of the bone flap: Brun. Sutures: Brun" [Dr. M. Brun, Vincent's assistant].

## COMMENTARIES

**Figure 2:**
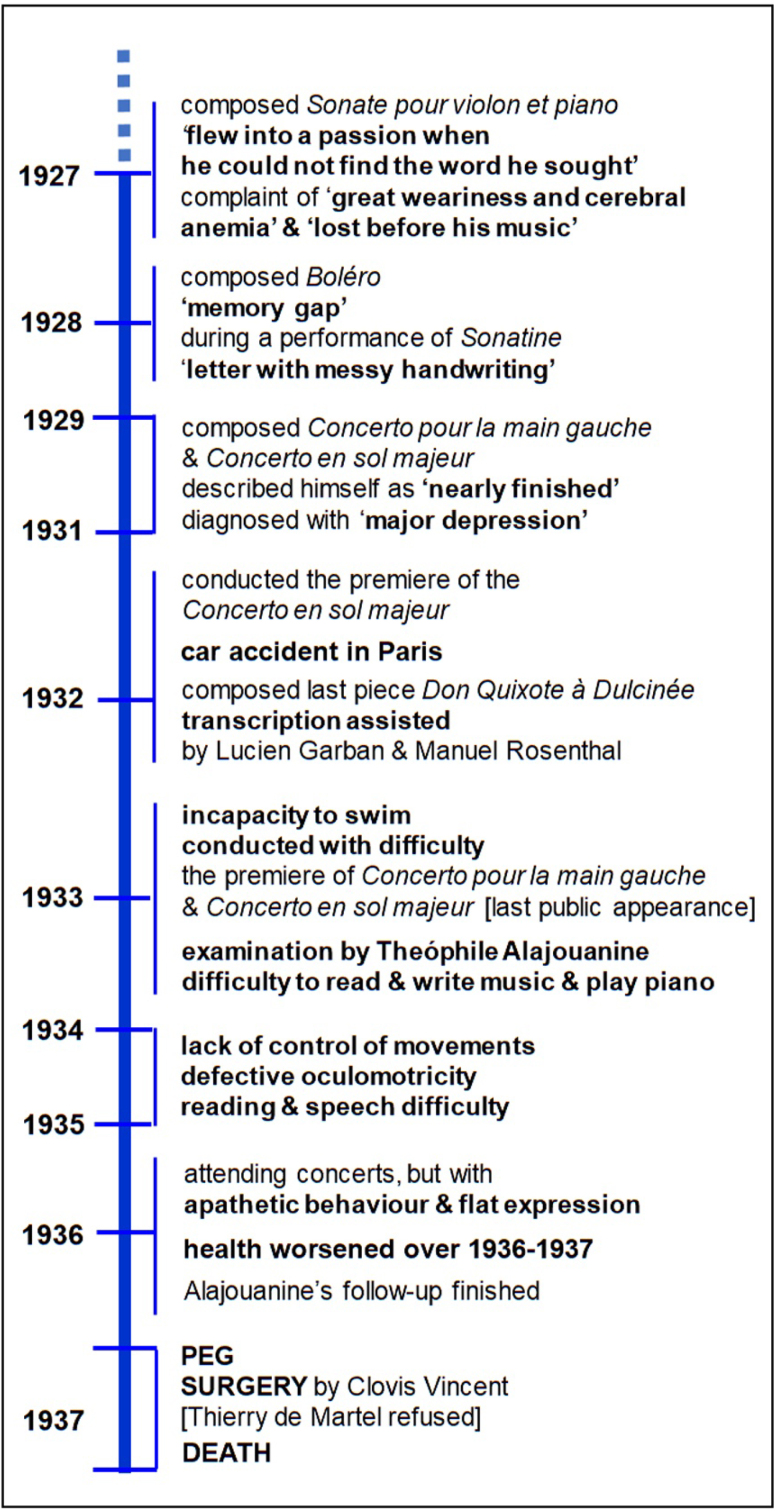
Timeline of the major milestones of the last ten years of Maurice Ravel's life^2,3,9,10^.

Since the initial onset of his disease in 1927, until his death in 1937, Ravel's verbal and musical language skills had progressively deteriorated, as depicted in the timeline of the last decade of his life (Figure 2)^2,3,9,10^. After thorough examinations, a contested neurosurgical intervention was proposed and performed by Clovis Vincent.

Many criticisms were leveled towards Vincent's surgery, at that time until today. It is true that the decision for a neurosurgical intervention occurred 86 years ago, with relatively scarce knowledge on such questions at the time. It should be acknowledged that Vincent had had relatively good neurosurgical training, as he initiated his career in this field in 1928 after visiting with renowned neurosurgeons in the United States, and certainly acquired some knowhow in gaseous encephalography (ventriculography and PEG), as he published papers about this subject (1933–1934)^15–17^.

Ravel was submitted twice to PEG, and except for the cited bilateral hydrocephalus, no further description was found. Serial photographs that were published showed few comparable views. The last studio portrait of Ravel (1935) shows prominent frontal bones, not beyond normal limits, while earlier pictures show no obvious signs of hydrocephalus, and no measurement of the head was found^3^. Thus, "hydrocephalus" was only an impression.

The craniotomy failed to substantiate the hypothesis that hydrocephalus or an expansive lesion might be the cause of Ravel's symptoms. It is necessary to underline that Vincent performed the craniotomy on the right side, arguing: "…to avoid damage to the dominant hemisphere…", and "…the ventricular dilatation was not due to a true atrophy, that it had increased with age, and that an operation might prevent progression…", and also "…the right hemisphere, which in his opinion was no longer compensating for deficiencies of the left, had to be inflated…". Weak arguments, in present-day views. The neurosurgical intervention proved useless, certainly precipitating Ravel's death^2,3,10,14,15^.

The surgical protocol (Box 2) cites "…Slack brain…Puncture of the right lateral ventricle: cerebral fluid escapes only on pressure…", suggesting that the hydrocephalus was either normo- or hypotensive, and excluding a possible expansive lesion. Furthermore, Vincent noted "…gyri separated by oedema…" A contradictory statement, considering that oedema means a tissular swelling [of the gyri], with narrowed or obliterated sulci, and not separated. Vincent possibly understood "oedema" as a collection of fluid inside dilated sulci, between the [atrophied] gyri^2,14^. Two conclusions can be extracted: normo- or hypotensive hydrocephalus, with separated [atrophied] gyri, probably was an *ex vacuo* ventricular dilatation, and also, if the right side showed atrophic changes, in the absence of other findings, the opposite side certainly would show similar changes, making the condition compatible with diffuse cerebral atrophy.

Considering Ravel's case, a PEG, if well-performed, could have shown a dilated ventricular system (with a deformation or deviation if some expansive lesion [tumor or hematoma] existed), and enlarged basal and convexity subarachnoid spaces (in case of a diffuse atrophic pattern or of a localized left-sided atrophy, degenerative or post-traumatic [considering an eventual sequel left by the car accident]). However, only bilateral dilated ventricles were described (probably symmetric), perhaps referring merely to the lateral ventricles, without further information. Thus, a well-executed PEG could have precluded the need for surgical intervention. With the evidence available at the time, de Martel refused to perform and advised against the surgery^3,10^. Additionally, Ravel's apparently disproportionately large head may have been an idiosyncratic characteristic, or possibly the result of a compensated chronic hydrocephalus, without clinical relevance on his symptoms.

Alajouanine performed his musical assessment prior to development of modern amusia testing^18^. However, his examination of Ravel was meticulous, which revealed an aphasic syndrome related to verbal and musical language, and ideomotor apraxia, which impaired his ability to read and write music, play the piano, and conduct an orchestra. Since Ravel was right-handed, this suggested that the localization of his verbal language function was most likely the left hemisphere of the brain, circumscribed to the left inferior frontal gyrus [Broca's area] (verbal expression), and the left parietal lobe (angular gyrus) (agraphia), and ideomotor apraxia^19^.

Musical language does not reveal a clear hemispheric lateralization, and the neuroanatomical substrates underlying its processing present an inter-hemispheric fragmented system. It seems that music processing is based on widely distributed, but locally specialized, subsystems^20,21^. There may be partial neuroanatomical overlap of the musical and verbal domains, and degeneration in such regions may lead to dysfunction, as in Ravel's case, who presented with mixed verbal and musical impairment. However, there may be neuroanatomical dissociation between both languages, where the loss of spoken language is not necessarily accompanied by loss of musical abilities^21^. Thus, musical processing is in part related to the left hemisphere, specifically the angular gyrus (e.g., musical alexia and agraphia); however, the right hemisphere also participates in such processing (relatively preserved melodic line and instrument playing)^21^. Such considerations are endorsed by what is known currently about aphasia and amusia (Box 3)^11,19–25^, affecting Ravel's verbal expressive language abilities (motor aphasia), and also a marked amusia, characterized by impairment of the ability to write and express music (musical alexia with agraphia, ideomotor apraxia), but with preserved ability to appreciate music.

Box 3 **Aphasia and amusia syndromes.**

**Acquired aphasia syndromes**

**Broca's aphasia:** due to a lesion in the left inferior frontal gyri (in most right-handed individuals) that leads to agrammatic, effortful speech, but comprehension is mostly preserved^19^.

**Wernicke's aphasia:** due to a lesion in the left inferior parietal lobe that leads to effortless speech, with significant impairment in comprehension^19^.

**Gerstmann's syndrome (component):** verbal agraphia^19^.

Alajouanine's diagnosis was Wernicke's aphasia^11^. However, Ravel's verbal comprehension, according to the neurologist, appears to have remained intact, while the expression was partially impaired (oral and graphic)^11^. Thus, his language disorder, based on the information available, could be a mixed one, with a motor component (left inferior gyrus [Broca's]), and a Gerstmann's (left angular gyrus) component. He presented ideomotor apraxia (left parietal lobe).

**Aquired amusia syndromes**

Several acquired amusia syndromes have been described, including expressive amusia, receptive amusia, amnesic amusia, musical alexia, musical alexia with agraphia, musical agraphia, and instrumental amusia^21,22^.

**Expressive amusia:** impaired singing, whistling, and humming, usually due to dysfunction of the right frontal and temporal lobes^22^.

Not evident with Ravel^11^.

**Receptive amusia:** difficulty to distinguish between different melodies, usually due to bilateral, or unilateral (left or right) lesions in the temporal and parietal lobes^22–24^.

Ravel maintained his ability to recognize different melodies and, hence, unlikely to have had receptive amusia^11^.

**Musical alexia:** difficulty to read musical scores, due to lesion in the left occipital and temporal lobes^22^.

Ravel had difficulty reading musical notes^11^.

**Amnesic amusia:** difficulty in recognizing familiar melodies, due to lesions mainly of the left hemisphere^20,22,23^.

Not evident with Ravel^11^.

**Oral expressive amusia:** impairment of singing, whistling, and humming, due to lesion of the right frontal and temporal lobes^22^.

Ravel exhibited mild oral expressive amusia, as he needed to be cued^11^.

**Musical agraphia:** impairment in the ability to write musical scores, due to lesion in the proximity of the left intraparietal sulcus^22,25^.

Ravel could not write his music^11^.

**Instrumental (apraxic) amusia:** difficulty in playing a musical instrument, having previous musical training, due to lesion in diverse bilateral regions, cortical and subcortical^21,22^.

He presented difficulty with playing the piano^11^.

**Musical alexia with agraphia:** difficulty in reading and writing musical scores, due to lesion in the left angular gyrus^20,23^.

Ravel presented such impairment.

Alajouanine's observations have provided the most direct observations of signs and symptoms of Ravel's illness. Many authors, based on the report of his examination and conclusions, proposed a number of diagnostic hypotheses of a pre-senile neurodegenerative condition to account for his symptoms, primarily indicating a subform of frontotemporal lobar degeneration and its allied disorders^21^ (Table 1)^1–4,8,9,11,12,26–29^. However, due to the lack of a post-mortem examination, Ravel's brain disease is doomed to remain a mystery.

**Table 1:** Some suggested diagnoses to account for Ravel's neurological illness.

Source	Year	Diagnosis
Alajouanine^11^	1948	Cerebral atrophy
Dalessio^26^	1984	Alzheimer's disease
Henson^3^	1988	Cerebral degeneration
Baeck^9^	1996	Primary progressive aphasia/ corticobasal degeneration
Alonso and Pascuzzi^1^	1999	Frontotemporal dementia
Amaducci et al.^12^	2002	Primary progressive aphasia/ corticobasal degeneration
Otte et al.^27^	2003	Traumatic brain injury
Cardoso^8^	2004	Tauopathy (Pick's complex)
Seeley et al.^28^	2008	Primary progressive aphasia/ corticobasal degeneration
Warren and Rohrer^29^	2009	Primary progressive aphasia/ corticobasal syndrome
Kanat et al.^2^	2010	Traumatic brain injury
Cavallera et al.^4^	2012	Primary progressive aphasia/ corticobasal degeneration

In conclusion, Ravel's disease caused a progressive impairment of his verbal and musical language domains. After repeated neurological and neuroradiological examinations, he underwent a neurosurgical intervention that, besides confirming the presence of an already known hydrocephalus, offered no further information. As autopsy was not permitted, there was no neuropathological substantiation of his underlying condition. Considering the progressive nature of his illness, many authors have speculated the presence of a neurodegenerative disease, particularly pertaining to a subform of the frontotemporal lobar degeneration complex and its allied disorders.
